# The Impact of Obesity on Childbirth Expectations

**DOI:** 10.1111/jmwh.13685

**Published:** 2024-09-09

**Authors:** Tamara A. Messer, Fabienne Blank, Jean Anthony Grand‐Guillaume‐Perrenoud, Evelyne M. Aubry

**Affiliations:** ^1^ Department of Health Professions Bern University of Applied Sciences Bern Switzerland

**Keywords:** birth, body mass index, labor, midwifery, obesity, perinatal care, pregnancy, survey

## Abstract

**Introduction:**

Positive childbirth expectations are crucial for fostering a positive labor experience and enhancing the health and well‐being of both the woman and her newborn. However, the impact of obesity on childbirth expectations remains underexplored. We aim to assess childbirth expectations in women living with obesity to enhance perinatal care tailored to their specific needs.

**Methods:**

Using an adapted version of the Childbirth Expectation Questionnaire (CEQ) in a nationwide online survey, we assessed expectations on childbirth of pregnant women living in Switzerland. We performed one‐way analysis of variance and independent *t* tests to analyze associations between childbirth expectations and women's characteristics such as body mass index (BMI). Binomial logistic regressions estimated the likelihood of positive birth expectations occurring based on individual and contextual factors.

**Results:**

In total, 961 pregnant women responded to the CEQ through social media. Increased BMI was associated with lower mean scores in overall birth expectations (*P* = .008), whereas women accompanied by midwives during pregnancy showed significantly increased mean scores (*P* < .001). Regression analysis revealed that women living with obesity were less likely to have positive expectations for their upcoming childbirth when compared with others (adjusted odds ratio [aOR], 0.63; 95% CI, 0.42‐0.95; *P* = .027). Conversely, midwifery care and plans for nonclinical births were associated with increased positive childbirth expectations (aOR, 3.65; 95% CI, 2.11‐6.32; *P* < .001 and aOR, 4.77; 95% CI, 3.37‐6.74; *P* < .001, respectively).

**Discussion:**

Women living with obesity exhibited significantly lower childbirth expectations compared with other women, impacting birth outcomes and satisfaction. Midwife involvement correlated with more positive expectations, emphasizing their role in improving women's realistic expectations and fostering well‐being. Enhanced accessibility to models of care with midwifery continuity may be a crucial factor in promoting positive expectations among women living with obesity.

## INTRODUCTION

Childbirth is a complex and overwhelming life event for all expectant women.^1^ Anticipating and setting childbirth expectations before this pivotal moment can serve as a valuable preparatory measure, facilitating both mental and physical readiness. Subsequently, women tend to use preconceived notions as a benchmark against which they evaluate their actual birth experience.^2^ The relationship between a woman's childbirth expectations and her actual experience can influence her evaluation of the event and impact her satisfaction with the whole birth process.^3^ Research has shown that increased similarity between childbirth expectations and the actual experience may positively affect women's ability to cope more successfully with labor.^4^ Therefore, positive childbirth expectations foster a positive experience of labor and thus contribute to increased health and well‐being for both the woman and her newborn.^3^, ^5^

Previous research has indicated that childbirth expectations have personal and sociocultural variations and will differ depending on past experiences, actual support, and information, as well as women's physical and emotional status.^6^, ^7^, ^8^ Women with limited access to education, of low socioeconomic status, or of younger age hold significantly fewer positive expectations for childbirth experiences compared with other pregnant women.^9^, ^10^, ^11^ Additionally, women with complicated pregnancies may not have their expectations met for a physiologic labor and birth free of medications and medical interventions. Hence, they may fail to adjust their childbirth expectations to actual circumstances, resulting in negative perceptions of labor, increased anxiety, and difficulties coping.^12^

Obesity is a common risk factor for complications during pregnancy and birth.^13^, ^14^ Women living with obesity (body mass index [BMI] ≥30) are at a higher likelihood of experiencing weight‐related morbidities, such as gestational diabetes, and pregnancy‐related hypertensive disorders.^13^ These conditions often lead to increased medical interventions during pregnancy, labor, and birth such as induction of labor or operative or cesarean birth, leaving expectations for physiologic childbirth unmet.^13^, ^14^, ^15^ Medicalized treatment and risk‐focused care, including increased surveillance and monitoring during labor, can contribute to heightened fear and, consequently, have further negative implications on birth expectations.^16^ Furthermore, women living with obesity often encounter discrimination and weight‐biased treatment from health care providers.^17^ Such negative attitudes from the perinatal care team, combined with the stigma associated with obesity, can influence women's perception and expectations of the quality of care.^18^ To deliver high‐quality birth care and improve childbirth experiences for pregnant women living with obesity, it is necessary to address more than risk reduction interventions.^19^ Support for positive childbirth expectations is also necessary to increase satisfaction and well‐being for this at‐risk population.

**QUICK POINTS**

✦ Positive childbirth expectations foster a positive experience of labor and birth and thus contribute to increased health and well‐being for both the woman and her newborn.
✦ Women living with obesity tend to have less positive childbirth expectations, potentially due to anticipating more interventions and increased anxiety about excessive pain.
✦ Limited access to alternative care options and settings for labor and birth could be a contributing factor to lower childbirth expectations of women living with obesity.

Although many studies evaluate women's experiences and satisfaction with current birth care, little is known about the childbirth expectations of women living with obesity.^20^, ^21^, ^22^, ^23^, ^24^ Childbirth expectations influence the motivation and efforts of women in achieving a positive childbirth experience. Moreover, knowing what women living with obesity expect for their upcoming births may be important first‐step information for antenatal caregivers to consider toward providing more appropriate childbirth preparation. Therefore, this study aimed to investigate the childbirth expectations of pregnant women living with obesity and whether their expectations differ from those of other women. By further identifying factors that play a role in influencing expectations, we explore which aspects could improve the quality of perinatal care.

## METHODS

### Participants and Data Collection

Pregnant women living in Switzerland and speaking German were anonymously recruited using social media advertisements between January and May of 2022. Lime Survey (www.limesurvey.com) was used to administer the questionnaire examining childbirth expectations. Participants were women older than 18 years old, pregnant, residents of Switzerland, and able to complete the online questionnaire in German. Women interested in participating in the anonymous online survey received comprehensive written study information at the beginning of the survey. By clicking the “Next” button to enter the survey, they indicated their willingness to voluntarily take part in this study, providing informed consent for an anonymous online survey. We acknowledge that not all individuals capable of giving birth identify as female. For the purposes of this study, the term *women* will be used to refer to women and gender‐diverse people capable of giving birth.

### Measures

#### Demographics

Participants provided self‐reported data on their weight and height immediately prior to pregnancy, which was then used to compute their prepregnancy BMI per standard calculation.^25^ Further, the participants were asked to provide information about their sociodemographic and perinatal characteristics such as age, education level, gestational age, parity, BMI, pregnancy complications, pregnancy care provider, and planned care provision during birth. The guidelines for reporting results of internet E‐surveys (Checklist for Reporting Results of Internet E‐Surveys) were followed (see Supporting Information: Checklist S1).

#### Childbirth Expectations

We used the modified version of the Childbirth Expectation Questionnaire (CEQ).^10^, ^26^, ^27^, ^28^, ^29^ The original CEQ consists of 35 items rated on a 5‐point Likert scale, ranging from strongly disagree (1) to strongly agree (5).^26^ The following 4 subscales reflect major areas of childbirth expectations: *coping and pain* (11 items), *nursing support* (8 items), *partner or coach* (7 items), and *intervention* (9 items). For instrument adaptation, a series of steps were taken (see Supporting Information: Table S1). First, the original instrument was translated to German and back‐translated to English, and different versions of translations were compared and harmonized to ensure conceptual equivalence. Thereafter, 9 pregnant women living with obesity and 9 experts in the field of obstetrics and midwifery were interviewed for a cognitive debriefing and to assess the relevance of the original items using the content validity index (CVI) (see Supporting Information: Text S1).^30^ A psychometric test was conducted including the total sample of 961 pregnant women (see Supporting Information: Text S1). Principal component analysis revealed the component structure of the instrument, with Bartlett's test and Kaiser‐Meyer‐Olkin Measure indicating the suitability of the analysis. Components were determined based on eigenvalues, with a criterion of eigenvalue > 1 and Scree plot analysis. Items with low component loadings were excluded, and Cronbach's α was computed to assess scale reliability.

### Statistical Analysis

Using the statistical program SPSS 28, comparisons were made among different groups of women based on various characteristics (BMI, pregnancy complications, pregnancy care provider, planned care provision during birth, and education level) regarding the total mean score and scores for the 3 components. These comparisons were carried out using one‐way analysis of variance (ANOVA) and independent *t* tests. Q‐Q plots were used to graphically determine normality. In the case of a statistically significant difference between the groups, Bonferroni post hoc tests were conducted to analyze which groups differed. The Levene test was conducted to test the assumption of homogeneity of variance.^31^ The level of significance was fixed at *P* < .05.

Using binomial logistic regression, odds ratios were estimated with 95% CIs to determine the likelihood of positive birth expectations occurring based on women's characteristics. The mean scores of the items measuring childbirth expectations were dichotomized (positive and negative childbirth expectations) and used as the dependent variable. Positive childbirth expectations were defined as means scores of ≥4.0 or ≤2.0 for reversed items. Odds ratios were further adjusted for BMI, pregnancy complications, pregnancy care provider, planned care provision during birth, and education level.

### Ethical Approval

The project was not subject to formal ethical approval as it did not fall under the Swiss Human Research Act (2011), Art. 2, para. 1.^32^

## RESULTS

### Demographics

Using the adapted CEQ, 961 pregnant women living in Switzerland made up the final sample. The mean (SD) age was 32.27 (3.8) years. Notably, nearly half were in their third trimester of pregnancy. Approximately 12.3% of the participants had a BMI exceeding 30 kg/m^2^. Obstetricians were actively involved in the prenatal care of the majority of participants, and 63.6% of the women had planned for obstetrician‐led births. In terms of education, a substantial 652 (67.8%) of the women held a university degree or an equivalent qualification (Table 1).

**Table 1:** Sociodemographic and Perinatal Characteristics of 961 Women Who Participated in the Survey

Characteristics	Value
**Age, mean (SD), y**	32 (3.8)
**Gestational age, n (%), wk**	
<12	274 (28.6)
12‐28	280 (29.1)
>28	407 (42.3)
**Body mass index, n (%)**	
<18.5	42 (4.4)
18.5‐25	592 (61.6)
25‐30	209 (21.7)
>30	118 (12.3)
**Pregnancy complications, n (%)**	
Gestational diabetes	36 (3.7)
Hypertension	15 (1.6)
Hyperthyroidism	57 (5.9)
Anemia	47 (4.9)
Mental illness	12 (1.2)
Premature contractions	28 (2.9)
**Pregnancy care provider, n (%)**	
Obstetrician	460 (47.9)
Midwife	118 (12.3)
Both	393 (39.9)
**Planned care provider during birth, n (%)**	
Obstetrician‐led	612 (63.6)
Midwife‐led	331 (34.5)
**Education, n (%)**	
Lower (primary, secondary)	309 (32.1)
Higher (postsecondary)	652 (67.8)

The cognitive interviews and content validity assessment of the original CEQ led to a 30‐item questionnaire with a CVI of .93, indicating that the items were appropriate for measuring childbirth expectations. After examining the distribution of items and drawing from previous scales,^10^, ^26^, ^27^, ^28^, ^29^ the components were labeled as follows: component 1 was termed “support and informed choice” (items 1‐10, a total of 10 items), component 2 was “pain and distress” (items 11‐20, a total of 10 items), and component 3 was “medical interventions” (items 21‐30, a total of 10 items). These 3 components collectively explained 39.1% of the total variance. The Cronbach's α coefficient was 0.85 for the whole CEQ (see Supporting Information: Table S1).

### Differences in Childbirth Expectations as a Function of Women's Characteristics

The results from the ANOVA and independent *t* tests revealed that positive childbirth expectations among pregnant women living with obesity (BMI >30 kg/m^2^) were significantly lower compared with women with a BMI less than or equal to 30 kg/m^2^ (mean [SD] 4.04 [0.33] vs 4.14 [0.36]; *P* = .017). Specifically, women with higher BMI displayed reduced mean (SD) scores in the components pain and distress and medical interventions (2.92 [0.67] vs 3.15 [0.63]; *P* = .002 and mean [SD] 3.14 [0.58] vs 3.37 [0.63]; *P* = .002 respectively). These results indicate that women do not have high expectations for effective pain and distress management, or for experiencing a birth free of medical interventions. Overall, women whose pregnancies were overseen by midwives scored higher in all 3 subscales, indicating higher expectations for positive childbirth experiences in contrast with those who received exclusive care from obstetricians (mean [SD] 4.63 [0.29] vs 4.47 [0.35]; *P* < .001; mean [SD] 3.48 [0.59] vs 3.01 [0.60], *P* < .001 and mean [SD] 3.78 [0.61] vs 3.09 [0.54]; *P* < .001). Additionally, women who intended to give birth in a midwife‐led setting reported positive childbirth expectations concerning support received, informed choice, and medical interventions during childbirth (mean [SD] 4.62 [0.30] vs 4.48 [0.37] *P* = .006). No significant differences were observed in childbirth expectations based on experienced pregnancy complications and education levels (Table 2).

**Table 2:** Total and in Subscale Means Scores on the Childbirth Expectation Questionnaire of Pregnant Women Living in Switzerland

Characteristics	Total	Support and Informed Choice	Pain and Distress	Medical Interventions
**BMI, mean (SD)** ^a^				
<18.5	4.12 (0.38)	4.51 (0.38)	3.05 (0.67)	3.33 (0.65)
18.5‐25	4.14 (0.36)	4.53 (0.35)	3.15 (0.63)	3.37 (0.63)
25‐30	4.08 (0.32)	4.53 (0.33)	3.09 (0.62)	3.18 (0.55)^b^
>30	4.04 (0.33)^b^	4.48 (0.33)	2.92 (0.67)^b^	3.14 (0.58)^b^
**Statistics**				
*P* Value^c^	.008	.56	.009	<.001
Effect (η^2^)	0.012	0.002	0.014	0.025
**Pregnancy complications,** ^d^ **mean (SD)** ^a^				
Yes	4.07 (0.35)	4.50 (0.39)	3.10 (0.67)	3.20 (0.65)
No	4.13 (0.37)	4.53 (0.33)	3.11 (0.63)	3.32 (0.60)
**Statistics**				
*P* Value^e^	.372	.056	.263	.687
Effect (Cohen's d)	0.35	0.34	0.64	0.61
**Pregnancy CP, mean (SD)** ^a^				
Obstetrician	4.01 (0.33)	4.47 (0.35)	3.01 (0.60)	3.09 (0.54)
Midwife	4.35 (0.32)^b^	4.63 (0.29)^b^	3.48 (0.59)^b^	3.78 (0.61)^b^
Both	4.18 (0.35)^b^	4.56 (0.34)^b^	3.12 (0.64)^b^	3.41 (0.59)^b^
**Statistics**				
*P* Value^c^	<.001	<.001	<.001	<.001
Effect size (η^2^)	0.11	0.027	0.054	0.143
**Planned care provision during birth, mean (SD)** ^a^				
Obstetrician‐led	4.01 (0.33)	4.48 (0.37)	3.00 (0.63)	3.00 (0.51)
Midwife‐led	4.35 (0.30)	4.62 (0.30)	3.38 (0.60)	3.31 (0.57)
**Statistics**				
*P* Value^e^	.729	.006	.57	.021
Effect (η^2^)	0.33	0.34	0.61	0.54
**Education,** ^f^ **mean (SD)** ^a^				
Lower	4.11 (0.33)	4.51 (0.32)	3.20 (0.63)	3.28 (0.58)
Higher	4.12 (0.37)	4.30 (0.35)	3.06 (0.63)	3.30 (0.63)
**Statistics**				
*P* Value^e^	.05	.451	.528	.057
Effect (Cohen's d)	0.35	0.34	0.63	0.62

Abbreviations: BMI, body mass index; CP, care provider.

a 5‐point Likert scale with anchors of 1 = strongly disagree and 5 = strongly agree. *P* values < .05 are significant.

b Bonferroni post hoc: total (BMI) 18.5‐25 vs >30, *P* = .017; medical intervention (BMI) 18.5‐25 vs 25‐30, *P* < .001, 18.5‐25 vs >30, *P* = .002; pain and distress (BMI) 18.5‐25 vs >30, *P* = .002; support and informed choice CP physician vs midwife, *P* < .001, physician vs both, *P* = .001; total CP physician vs midwife, *P* < .001, physician vs both, *P* value < .001, midwife vs both, *P* < .001; medical interventions CP physician vs midwife, *P* < .001, physician vs both, *P* < .001, midwife vs both, *P* < .001; support and informed choice CP physician vs midwife, *P* < .001, midwife vs both, *P* < .001.

c Welch analysis of variance is used to compute *P* values.

d Pregnancy complications: gestational diabetes, hypertension, hyperthyroidism, anemia, mental illness, premature contractions.

e Independent *t* test is used to compute *P* values.

f Education: lower (primary, secondary), higher (postsecondary).

### Risk Factors Intervening With Positive Childbirth Expectations

In Table 3, odds ratios were presented for expecting positive childbirth experiences (dichotomized into positive ratings ≥4 and negative ratings ≤3) based on combinations of women living with obesity, having pregnancy complications, receiving care provided by midwives during pregnancy, intending to give birth in a midwife‐led unit, and exhibiting higher education. Obesity and complicated pregnancies were associated with a higher incidence of negative childbirth expectations (adjusted odds ratio [aOR], 0.63; 95% CI, 0.42‐0.95; *P* = .027 and aOR, 0.68; 95% CI, 0.48‐0.95, *P* = .025). This was largely attributed to the increased likelihood of women expecting a medicalized approach to their labor and birth.

**Table 3:** Factors Associated With Positive Childbirth Expectations in Total and Subscales

	Total		Support and Informed Choice		Pain and Distress		Medical Interventions	
Characteristics	aOR (95% CI)	*P* value	aOR (95% CI)	*P* value	aOR (95% CI)	*P* value	aOR (95% CI)	*P* value
BMI >30 kg/m^2^ ^a^	0.63 (0.42‐0.95)	.027	0.58 (0.26‐1.27)	.173	0.88 (0.43‐1.80)	.721	0.46 (0.24‐0.87)	.017
Pregnancy complications^b^	0.68 (0.48‐0.95)	.025	0.85 (0.42‐1.73)	.654	1.01 (0.59‐1.74)	.968	0.49 (0.30‐0.81)	.005
Midwife as care provider during pregnancy^c^	3.65 (2.11‐6.32)	<.001	6.69 (0.91‐49.17)	.062	2.73 (1.59‐4.68)	<.001	5.44 (3.54‐8.35)	<.001
Midwife‐led care during birth^c^	4.77 (3.37‐6.74)	<.001	1.81 (0.91‐3.60)	.092	2.85 (1.81‐4.49)	<.001	9.16 (6.11‐13.75)	<.001
Higher education^d^	1.08 (0.81‐1.44)	.614	1.79 (0.90‐3.58)	.098	0.66 (0.42‐1.04)	.074	1.15 (0.78‐1.68)	.484

Abbreviations: aOR, adjusted odds ratio; BMI, body mass index.

a Binominal logistic regression model was controlled for age, gestational age, parity, pregnancy complications, education.

b Binominal logistic regression model was controlled for age, gestational age, parity, BMI, and education (pregnancy complications: gestational diabetes, hypertension, hyperthyroidism, anemia, mental illness, and premature contractions).

c Binominal logistic regression model was controlled for age, gestational age, parity, BMI, pregnancy complications, and education.

d Binominal logistic regression model was controlled for age, gestational age, parity, BMI, and pregnancy complications.

Conversely, women receiving prenatal care from midwives, or planning to give birth in a midwifery‐led setting, were 3 to 4 times more likely to have positive childbirth expectations than women receiving standard care (aOR, 3.65; 95% CI, 2.11‐6.32; *P* < .001 and aOR, 4.77; 95% CI, 3.37‐6.74; *P* < .001). No significant association between positive childbirth expectations and levels of education was identified (aOR, 1.08; 95% CI, 0.81‐1.44; *P* = .614).

## DISCUSSION

A major finding of this study indicated that women living with obesity expected childbirth to be overall less positive than those reporting a BMI of less than or equal to 30 kg/m^2^. In particular, women living with obesity expected more difficulties in coping with pain and distress during labor and were less likely to have positive expectations regarding their need for medical interventions. In the literature, we found similar results regarding pregnant women at increased risk of pregnancy and birth complications. These women had fewer positive expectations regarding their upcoming childbirth.^12^ The findings further suggested that women who were aware of the potential risk of a complicated birth interpreted labor as a threat, resulting in a higher level of anxiety and decreased faith in their coping abilities, which negatively impacted childbirth expectations.^12^ These findings could align with current perinatal care for women living with obesity, which places a strong emphasis on increased risk for perinatal complications. Focusing on the promotion of medical safety for these women then leads to increased fear of complications that occur during childbirth.^13^, ^14^, ^33^ Furthermore, when validating the CEQ questionnaire, specific questions related to discrimination and disregard while awaiting labor were associated with increased distress and higher pain expectations. This could indicate that women living with obesity perceive pain and distress as being associated with birth‐specific conditions. In addition, anxiety and concerns about how they might be treated by others may arise. For instance, women with obesity may fear having interactions with professionals who are not aware of their needs and who might judge them or discriminate against them.^34^ Therefore, it would be preferable for care providers to explore women's expectations and fears of judgment or exclusion and provide accurate support to ensure a safe care environment.

Given the inverse relationship between medicalized or biased care and childbirth expectations, a shift toward more holistic models of birth may thus positively impact expectations regarding birth experience.

Midwifery prenatal care options were attributed to significantly higher scores of positive childbirth expectations in all subscales of this study. Furthermore, receiving care from midwives results in an increased likelihood of experiencing positive childbirth expectations. Women in this sample associated the physician's presence with the medical intervention domain, whereas the midwife's presence clustered with support and informed choice. In Swiss hospitals, obstetricians are often only present for the birth of the newborn or when complications arise. Midwives, on the other hand, monitor and care for the women during the birth process.^35^ It is likely that women in our sample viewed the obstetrician as primarily involved in case of complications and interventions. Data from a large survey in California explored the role of midwives versus obstetricians from the perspective of women's reported birth experience.^36^ Women who received care from obstetricians during childbirth had an increased likelihood of undergoing multiple medical interventions, felt more pressured to opt for epidural analgesia, and had a lower sense of support in their decision‐making process compared with women in midwifery‐led care. Obstetricians may thus be perceived by women in this sample as competent providers available to ensure access to necessary medical interventions should complications arise. In turn, from women's perspectives, midwives' personal support and provision of information are expected and appreciated.^37^

Having continuous support from a midwife may provide emotional support, comfort, information, and advocacy, enhancing women's sense of control and reducing the need for medical interventions.^38^ However, alternative continuity of midwifery care models such as freestanding birth centers or home birth typically involve the care of women with lower risk of complications.^39^ These models are often inaccessible for women living with obesity given their *high‐risk* label.^40^ Various international guidelines assume that these women will give birth in hospitals, with intravenous access and continuous electronic fetal monitoring.^41^

To specifically increase the likelihood of positive childbirth expectations among women living with obesity, midwives and other perinatal care providers should consider overall health in addition to body weight measurements when discussing risks of complications. Particularly, midwives' support of a physiologic birth could improve the likelihood of expecting fewer medical interventions during labor and birth.^42^ Overall, improved childbirth expectations through better access to midwifery care may enhance women's motivation for various efforts, such as adherence to treatment and proactive health management during pregnancy, and increase maternal self‐efficacy in preparing for labor.^43^, ^44^ Moreover, increased positive expectations may lead to greater control during birth, greater satisfaction, and improved emotional well‐being.^43^

### Implication for Practice and Research

Obesity affects birth care and short‐ and long‐term outcomes for women and their newborns, constituting multifaceted challenges for health professionals. Additionally, it places a significant burden on health services.^45^ It is essential to gather a deeper understanding of potential mechanisms between maternal obesity and perinatal outcomes from a biopsychosocial perspective to facilitate optimal birth care for women living with obesity.

This study emphasizes that pregnant women with obesity have lower childbirth expectations, underscoring the importance of strategies to improve and meet these expectations to enhance the quality of care. Prioritizing awareness of their preferences and fears is essential for providing optimal and respectful birth care tailored to their specific needs.^46^

Midwives, nurses, and perinatal services face considerable challenges but also opportunities in providing and maintaining high‐quality care. The importance of high‐quality research to improve perinatal services to the health and well‐being of women living with obesity is increasingly recognized by researchers, policymakers, and clinical staff.^47^

The results of this study support the specific roles of midwives in providing birth care for women living with obesity. Midwives should ensure that childbirth care is individualized and needs‐oriented and includes support for long‐term perinatal and neonatal health.^48^ Strategies for improving positive childbirth expectations should be identified to enhance birth satisfaction. The psychometrically sound tool developed and implemented in this study for assessing childbirth expectations in this population can be used as an evaluation tool for the effectiveness of interventions to improve childbirth expectations and experiences.

### Strengths and Limitations

This study has some strengths and limitations that need to be acknowledged. The use of social media recruitment allowed us to access to a large and previously hard‐to‐reach population of women living with obesity. Although randomized sampling is considered necessary for generalization, social media recruitment offers several advantages over traditional methods, including shorter recruitment times, improved participant selection, and high privacy standards.^49^, ^50^ However, it is important to note that the study's generalizability is limited because it focuses exclusively on women living in Switzerland who use social media. Nevertheless, many young and middle‐aged women in Switzerland are active social media users. Studies have also shown that samples recruited via social media can be similarly representative to those obtained through other methods.^51^, ^52^ But it should be noted that women in this sample have an education level above the average in Switzerland. Although this study demonstrated no significant association between education level and childbirth expectations, in a sample in which there are more women of lower educational background, such an association cannot be excluded. Further research will be needed with a larger and more diverse sample to ensure the generalizability of the findings across different demographic groups and settings.

Another limitation to consider is that women's expectations were measured only once. Women, particularly in early pregnancy, may have changed their expectations throughout their pregnancy. Additional investigation will be necessary to explore how childbirth expectations may change throughout the individual experiences of women living with obesity. Finally, this study included a sample of pregnant women in Switzerland. Midwifery care models vary significantly between countries, which can influence women's expectations and experiences of childbirth. Differences in care practices and access may affect how childbirth is managed and perceived, potentially limiting the generalizability of the study's findings across different contexts.^53^

## CONCLUSION

We found women living with obesity to have significantly lower expectations for their childbirth experience.^9^ Presumably, this might be due to their high‐risk medical status, which could reduce confidence in their physical and psychological capacity to give birth. It is important to recognize the existence of childbirth expectations that generate anxiety, and fear of childbirth may lead to dissatisfaction and increase risks for a complicated birth.

Midwifery care appears to be a significant factor contributing to more positive childbirth expectations. Midwifery care models may help women living with obesity develop realistic expectations, trigger positive emotions, and promote birth satisfaction and women's well‐being. Enhancing positive childbirth expectations when caring for women living with obesity is crucial to ensure a respectful care environment and empower these women with self‐confidence.

## CONFLICTS OF INTEREST

The authors have no conflicts of interest to disclose.

## Supporting information


**Table S1**. Matrix with Component Loadings from Principal Component Analysis with Promax Rotation
**Text S1**. Supplementary Information about the Validation of the CEQ

**Checklist S1**. Guidelines for reporting results of internet E‐surveys (CHERRIES)
