# Supplementary material for: The Impact of Obesity on Childbirth Expectations
Source: J Midwifery Womens Health. 2024 Sep 9;70(1):96–103. doi: 10.1111/jmwh.13685 (PMC11803491; doi:10.1111/jmwh.13685)
Supplement: Supplementary file 1 — Table S1. Matrix with Component Loadings from Principal Component Analysis with Promax Rotation Text S1. Supplementary Information about the Validation of the CEQ [file JMWH-70-96-s002.docx]

# **Supplementary Data**

**Table S1.** Matrix with Component Loadings from Principal Component Analysis with Promax Rotation

**Item**

**Number** **Items**  **Component Loadings**

|  | **Component 1** Support and  informed choice | **Component 2** Pain and distress | **Component 3** Medical interventions |
| --- | --- | --- | --- |
|  | 0.63 |  |  |
| R | 0.57 |  |  |
| R | 0.57 |  |  |
| R | 0.56 |  |  |
|  | 0.54 |  |  |
|  | 0.52 |  |  |
| + | 0.49 |  |  |
| + | 0.46 |  |  |
|  | 0.42 |  |  |
| R | 0.40 | 0.71 |  |
| R |  | 0.71 |  |
| R |  | 0.69 |  |
|  |  | 0.69 |  |
|  |  | 0.60 |  |
|  |  | 0.51 |  |
|  |  | 0.48 |  |
|  |  | 0.46 |  |
| + |  | 0.44 |  |
| R |  | 0.43 |  |
| R |  |  | 0.76 |
| R |  |  | 0.74 |
|  |  |  | 0.69 |
| + |  |  | 0.69 |
| R |  |  | 0.69 |
| R |  |  | 0.65 |
| R |  |  | 0.61 |
|  |  |  | 0.58 |
| R |  |  | 0.42 |
| R |  |  | 0.41 |
|  | 0.71 | 0.81 | 0.75 |
|  | 10.4 | 13.0 | 15.7 |
|  | 10.4 | 23.4 | 39.1 |

With regard to my labor, I expect that

1. the midwives offer me encouragement
2. I will feel well taken care of with the midwife’s presence
3. the midwives will treat me with empathy
4. I will have the opportunity to actively participate in decision-making

my opinion and that of my partner/birth companion will be sought for all my major decisions

5

6 I will receive personal attention from the midwives 7 the doctors will treat me with empathy

8 I will always be informed in advance of routine treatments

I will feel comforted by the presence of my partner/birth companion

9

10 I will ask my partner/birth companion for support 11 I will be scared when I think about the pain of labor 12 I will be afraid of losing control

1. I will be afraid of feeling like a failure
2. I will worry about severity for labor pain 15 I will be embarrassed by my behavior 16 The pain of labor will be agonizing

17 I will be able to relax during labor 18 I will feel intense pain

1. I could be discriminated against
2. my plans for birth might be disregarded

many medical devices (heart sound monitoring, ultrasound, etc.) will be used

2121

I will be required to undergo routine procedures (like fetal

22

monitoring, vaginal examination, etc.) even if I don’t want them

1. I will use anesthetics and/or pain killer drugs
2. I will feel well taken care of with the doctor’s presence
3. I will want to have continuous fetal monitoring

I will deliver my baby without any medical intervention (Epidural anesthesia, induction of labor)

26

I will refuse to have any procedure (induction of labor, episiotomy etc.) I consider unnecessary

27

28 the doctors will make most of the decisions 29 forceps or suction cup will be used

the risk of having to give birth by caesarean section will be very low for me

30

Cronbach's alpha^a^ Variance explained (%)

Cumulative variance explained (%)

Abbreviation: R: revised item; +: new item

Component loadings < 0.40 are not shown.

^a^ Cronbach's alpha coefficient of 0.85 for the whole CEQ

Excluded items were: With regard to my labor, I expect that: The midwives will have only little time for me, I will experience severe but not unbearable pain, My partner/birth companion will feel quite helpless, I will avoid telling my partner/birth companion what I am feeling, I will be able to psychologically cope with labor, I will avoid asking the midwives for additional support, My partner/birth companion will look forward to our child, I will be immobilized by the pain of labor, My partner/birth companion will tell me what is going on.

**Text S1. Supplementary Information about the Validation of the CEQ**

*Content validity*

Content validity was tested by interviewing nine pregnant women living with obesity and nine experts in the field of obstetrics and midwifery for a cognitive debriefing and to assess the relevance of the original items using the content validity index (CVI).

For each item, the women and experts rated its clarity (not clear (0) / clear (1)) and relevance (1=not, 2=somewhat, 3=quite, 4=highly relevant) in relation to childbirth expectations (Polit et al., 2007). The CVI score was calculated by dividing the number of experts giving a rating of either 1 for clarity and either 3 or 4 for relevance with the total number of the experts.

During cognitive interviews, women in Switzerland consistently emphasized the importance of being informed and advised about procedures in advance. Additionally, weight stigmatization was perceived by women living with obesity as a significant factor influencing childbirth expectations. Consequently, items 8 and 19 were created and newly added to the questionnaire to incorporate these dimensions. Similarly, items 7 and 24 were added, but they focus on the view of the obstetrician instead of the midwife, akin to items 2 and 3.

*Psychometric testing*

Due to the limited number of cases in the subsample of women living with obesity (n = 118), the total sample of 961 pregnant women was used for the principal component analysis. We discovered the component structure among the items of the instrument and evaluated the contribution of each item to assess childbirth expectations of women living with obesity. The Bartlett's test (Chi-square = 11262.79, p < .001), the Kaiser-Meyer-Olkin Measure of Sampling Adequacy (KMO = 0.879), and the anti-image correlation (> 0.6) indicated that principal component analysis could be performed on the scale.

The extraction of components was performed using principal component analysis (varimax rotation). The number of components was determined based on the proportion of explained variance of a component (eigenvalues). The assessment was conducted using the Kaiser-Guttman criterion (eigenvalue > 1) as well as the Scree plot. Components with a minimum of three to five items per component were accepted, along with a variance of more than 10% per component (Tabachnick, 2007). Items with component loadings below 0.4 were excluded as they indicate that they do not measure the construct of "childbirth expectations" and thereby impair the internal consistency of the scale.

Cronbach's alpha was computed to evaluate the overall scale and subscale reliability.

*Results of the content validity and psychometric testing*

The cognitive interviews and content validity assessment of the original CEQ led to revisions of wordings in 17 items. Four new items were added, as they are perceived as missing by the interview participants (My partner/birth companion will look forward to our child, the doctors will treat me with empathy, I will feel well taken care of by the doctor’s presence, I will always be informed in advance of routine treatments, I could be discriminated against). Two items were removed due to non-relevance to interviewees (I will be immobilized by the pain of labor, my partner/birth companion will tell me what is going on). The remaining items were deemed relevant to pregnant women living with obesity. The CVI was . 93 indicating that the items were appropriate.

A principal component analysis was conducted, revealing potential solutions involving up to 9 components. Initially, a set of 35 items was entered into the principal component analysis (Gupton et al., 1991). Three items with maximum loadings below 0.3 were excluded (I will be able to psychologically cope with labor, I will avoid asking the midwives for additional support, my partner/birth companion will look forward to our child). Furthermore, the items "the midwives will have only a little time for me", "I will experience severe but bearable pain", "my partner/coach will feel somewhat helpless", and "I will avoid sharing my feelings with my partner/coach" exhibited loadings between 0.3 and 0.4, and cross-loadings to other components were under 0.2. Consequently, these items were also removed as they decreased Cronbach's alpha for their respective components (Table S1).

Upon removing these items and guided by the scree plots, in addition to considering the minimum number of 3-5 items and a minimum variance of 10% per extracted component, as well as ensuring acceptable reliability (α ≥ 0.7 per component), a 30-item scale with three-components emerged as the most suitable choice.

After examining the distribution of items and drawing from previous scales (Gupton et al., 1991; Kao et al., 2004; Martínez-Borba et al., 2022; Peñacoba-Puente et al., 2016; Xian Zhang, 14 C.E.) the components were labeled as follows: component 1 was termed "support and informed choice" (items 1-10, a total of 10 items), component 2 "pain and distress" (items 11-20, a total of 10 items), and component 3 "medical interventions" (items 21-30, a total of 10 items). These three components each and collectively explained 10.4%, 13%, 15.7% and of the variance, contributing to a total of 39.1%.

The internal consistency of all three components proved as satisfactory, with values above 0.7 (Table S1).

**References**

Gupton, A., Beaton, J., Sloan, J., & Bramadat, I. (1991). The development of a scale to measure childbirth expectations. *Can J Nurs Res*, *23*(2), 35–47.

Kao, B. C., Gau, M. L., Wu, S. F., Kuo, B. J., & Lee, T. Y. (2004). A comparative study of expectant parents ’ childbirth expectations. *J Nurs Res*, *12*(3), 191–202. https://doi.org/10.1097/01.jnr.0000387503.21749.0d

Martínez-Borba, V., Suso-Ribera, C., Catalá, P., Marín, D., & Peñacoba-Puente, C. (2022). Psychometric properties of the Childbirth Expectation Questionnaire in a sample of Spanish pregnant women. *Clínica y Salud*, *33*(1), 1–9. https://doi.org/10.5093/clysa2021a10

Peñacoba-Puente, C., Carmona-Monge, F. J., Marín-Morales, D., & Écija Gallardo, C. (2016). Evolution of childbirth expectations in Spanish pregnant women. *Applied Nursing Research: ANR*, *29*, 59–63. https://doi.org/10.1016/j.apnr.2015.05.017

Polit, D. F., Beck, C. T., & Owen, S. V. (2007). Is the CVI an acceptable indicator of content validity? Appraisal and recommendations. *Research in Nursing & Health*, *30*(4), 459–467. https://doi.org/10.1002/nur.20199

Tabachnick, R. ; B. G. ; and L. S. ; F. (2007). *Using Multivariate Statistics.* Pearson.

Xian Zhang, H. L. (14 C.E.). Childbirth expectations and correlates at the final stage of pregnancy in Chinese expectant parents. *International Journal of Nursing Science*, *1*(2), 151–156.
