# Supplementary material for: The Impact of Obesity on Childbirth Expectations
Source: J Midwifery Womens Health. 2024 Sep 9;70(1):96–103. doi: 10.1111/jmwh.13685 (PMC11803491; doi:10.1111/jmwh.13685)
Supplement: Supplementary file 2 — Checklist S1. Guidelines for reporting results of internet E‐surveys (CHERRIES) [file JMWH-70-96-s001.pdf]

Checklist for Reporting Results of Internet E-Surveys (CHERRIES)

Item Category	***Checklist Item***	***Explanation***
Design	Describe survey design	The study is a convenience sample. Pregnant women living in Switzerland and speaking German were anonymously recruited using social media advertisements between January and May of 2022. Lime Survey (www.limesurvey.com) was used to administer the questionnaire examining childbirth expectations.
	IRB approval	The project had not been subject to formal ethical approval (clarification of responsibility at the Ethics Committee for Research of the Canton of Bern, Switzerland, BASEC-No. Req-2021-00255) as it did not fall under the Swiss Human Research Act (2011), Art. 2, para. 1.
	Informed consent	Women interested in participating in the anonymous online survey received comprehensive written study information at the beginning of the survey. By clicking the 'Next' button to enter the survey, they indicated their willingness to voluntarily take part in this study, providing informed consent for an anonymous online survey.
	Data protection	The anonymously collected data was stored in the specific computer server from the Bern, University of Applied Sciences, Switzerland. Only specific personnel who signed a confidentiality agreement could view and analyze the data.

Development and pretesting	Development and testing	Before formal data collection, the CEQ questionnaire was translated and culturally adapted using cognitive interviewing. Furthermore, the content validity index was determined for each item. The questionnaire was pretested and validated using psychometric testing.

Recruitment process and description of the sample having access to the questionnaire	Open survey versus closed survey	Open survey. Participants could access the questionnaire over social media
	Contact mode	Initial contact was made by filling in the questionnaire via social media.
	Advertising the survey	We promoted the survey through the official account of online platforms.
	Web/E-mail	The survey was published on an online questionnaire platform (lime survey).
	Context	The commercial platform specializes in publishing online questionnaires. We only used it for data collection.
	Mandatory/voluntary	The survey was voluntary.
	Incentives	There were no incentives for participants to answer the survey.
	Time/Date	The data was collected between January and May of 2022.
	Randomization of items	The question order was not randomized.

	or questionnaires	
	Adaptive questioning	There were adaptive questions in the questionnaire so that respondents could jump past unnecessary questions based on their answers. For example, if the subjects answered that they gave birth by cesarean section, they did not answer the questions about vaginal births.
Survey administration	Number of Items	The questionnaire had 42 questions.
	Number of screens (pages)	6 screens.
	Completeness check	Technically, it was possible to do completeness checks before the questionnaire was submitted, and selecting a response option should be enforced.
	Review step	Respondents were allowed to go 'back' in the survey.

Response rates	Unique site visitor	N/A.
	View rate (Ratio of unique survey visitors/unique site visitors)	The survey is voluntary. The system cannot record the number of unique visitors, so the view rate cannot be calculated.
	Participation rate (Ratio of unique visitors who agreed to participate/unique first survey page visitors)	The system cannot record the number of people to fill in and the number of visitors, so the participation rate cannot be calculated.

	Completion rate (Ratio of users who finished the survey/users who agreed to participate)	This was a voluntary questionnaire. By default, the participants submit questionnaires on behalf of their consent to participate, so the completion rate cannot be calculated.

Preventing multiple entries from the same individual	Cookies used	Cookies were not used.
	IP check	The IP was not recorded to guarantee anonymous collection.
	Log file analysis	N/A
	Registration	N/A

Analysis	Handling of incomplete questionnaires	Incomplete surveys were not recorded and analyzed.
	Questionnaires submitted with an atypical timestamp	The system had no cache mechanism after exiting the questionnaire. Therefore, incomplete surveys were not recorded and analyzed.
	Statistical correction	Avoid the deviation of results by using appropriate statistical methods. For example, the dependent variable of this study was nonnormal distribution, so binary logistic regression was used instead of linear regression. In addition, the bias-corrected percentile Bootstrap test was used to extract 1,000 repetitions.
